# Surveillance of *Coxiella burnetii* Shedding in Three Naturally Infected Dairy Goat Herds after Vaccination, Focusing on Bulk Tank Milk and Dust Swabs

**DOI:** 10.3390/vetsci9030102

**Published:** 2022-02-24

**Authors:** Benjamin U. Bauer, Clara Schoneberg, T. Louise Herms, Martin Runge, Martin Ganter

**Affiliations:** 1 Clinic for Swine and Small Ruminants, Forensic Medicine and Ambulatory Service, University of Veterinary Medicine Hannover, Foundation, Bischofsholer Damm 15, 30173 Hannover, Germany; martin.ganter@tiho-hannover.de; 2 Department of Biometry, Epidemiology and Information Processing, WHO Collaborating Centre for Research and Training for Health in the Human-Animal-Environment Interface, University of Veterinary Medicine Hannover, Foundation, Bünteweg 2, 30559 Hannover, Germany; clara.schoneberg@tiho-hannover.de; 3 Lower Saxony State Office for Consumer Protection and Food Safety (LAVES), Food and Veterinary Institute Braunschweig/Hannover, Eintrachtweg 17, 30173 Hannover, Germany; louise.pruefer@laves.niedersachsen.de (T.L.H.); martin.runge@laves.niedersachsen.de (M.R.)

**Keywords:** bulk tank milk, dust swab, goat, longitudinal study, milking parlor, phase-specific serology, One Health, Q fever, vaccination, zoonosis

## Abstract

Q fever outbreaks on three dairy goat farms (A–C) were monitored after the animals had been vaccinated with an inactivated *Coxiella burnetii* phase I vaccine. The antibody response was measured before vaccination by serum samples with two *C. burnetii* phase-specific ELISAs to characterize the disease status. Shedding was determined by vaginal swabs during three kidding seasons and monthly bulk tank milk (BTM) samples. Dust swabs from one windowsill of each barn and from the milking parlors were collected monthly to evaluate the indoor exposure. These samples were analyzed by qPCR. The phase-specific serology revealed an acute Q fever infection in herd A, whereas herds B and C had an ongoing and past infection, respectively. In all three herds, vaginal shedders were present during three kidding seasons. In total, 50%, 69%, and 15% of all collected BTM samples were *C. burnetii* positive in herds A, B, and C, respectively. Barn dust contained *C. burnetii* DNA in 71%, 45%, and 50% of examined swabs collected from farms A, B, and C, respectively. The largest number of *C. burnetii* positive samples was obtained from the milking parlor (A: 91%, B: 72%, C: 73%), indicating a high risk for humans to acquire Q fever during milking activity.

## 1. Introduction

Q fever is a zoonotic disease caused by the obligate intracellular bacterium *Coxiella burnetii*. The pathogen has a high tenacity, with resistance to desiccation, heat, UV light, and numerous disinfectants [1,2]. The main reservoirs of the pathogen are cattle, sheep, and goats, which shed the bacteria mainly through birth products during abortion or normal parturition, but also through feces and milk [3,4]. Reproductive disorders are known in goats, with high abortion rates up to 90%, stillbirth, and weak kids [3,5,6]. The impact of *C. burnetii* on cattle health is still unknown and under debate [7], whereas sheep seem to be less affected, with low abortion rates of up to 5% [8].

Humans become easily infected with *C. burnetii* when inhaling contaminated aerosols and dust. The median infectious dose (ID50) was estimated at 1.5 bacteria and indicates the high infectivity of *C. burnetii* via aerosols [9]. In the Netherlands, drifting particulate matter from *C. burnetii* positive dairy goat farms led to the world’s largest Q fever epidemic, with more than 4000 reported human cases and an estimated number of about 40,000 infected individuals [10,11]. Furthermore, one ‘super-spreading’ ewe infected 299 visitors at a German farmers’ market [12]. In addition to inhalation, the consumption of raw milk contaminated with *C. burnetii* is considered a sporadic route of transmission [2,13]. Approximately 40% of infected humans show flu-like symptoms, such as fever, pneumonia, and hepatitis [14]. In the long term, up to 20% of patients with acute Q fever develop chronic fatigue syndrome [15], and affected patients with lesions of the cardio-vascular system suffer from endocarditis and vascular disease [14,16]. Seroprevalence among people within the European Union is highly diverse, and ranges from 1% in the general population, up to 83.8% in livestock veterinarians [17]. A *C. burnetii* seroprevalence of 3.1% was reported for humans in the United States [18]. In Australia, a seroprevalence of approximately 5% was determined in the rural population and in people living in metropolitan areas [19]. These results show that the risk of contracting Q fever is independent from the place of residence.

A particular characteristic of *C. burnetii* is its antigenic phase variation, leading to phase I (PhI) and phase II (PhII), which correlates with lipopolysaccharide changes [20,21]. These features are used for phase-specific serology to investigate the infection dynamics in goat flocks. The rise in IgG PhII antibodies without the appearance of IgG PhI are interpreted as a recently acquired infection [22,23]. Equal levels of IgG PhI and PhII represent an ongoing infection and occur approximately nine weeks post-infection [22]. The exclusive presence of IgG PhI antibodies outlined an infection in the past [24]. Nevertheless, phase-specific serology is still not used for routine diagnostic in veterinary medicine, despite it being a valuable tool for studying infection dynamics in goat herds [25].

Without control measures, *C. burnetii* may circulate in goat herds for several years [24,26,27]. To prevent Q fever outbreaks in livestock, an inactivated *C. burnetii* phase I vaccine has been licensed for cattle and goats in several European countries [28]. This vaccine significantly reduced abortion rates and excretion of the bacteria in naïve goats when given before mating [29]. Vaccination of pre-infected goats did not prevent vaginal shedding of *C. burnetii*, but reduced the amount of pathogen excretion and may also lower the number of shedders [30,31,32]. However, data about the long-term effect of *C. burnetii* phase I vaccine in naturally pre-infected goats on pathogen excretion are missing. Such information is essential for veterinary health care officials and other decision-makers to assess the risk of *C. burnetii* transmission to humans after a Q fever outbreak.

Bulk tank milk (BTM) samples have been successfully used to monitor the Q fever status of dairy goat herds [33,34,35]. Analyzing caprine BTM samples for *C. burnetii* by PCR revealed a wide range of detection rates in several countries, such as 16% in Belgium [35], 32.9% in the Netherlands [34], 54% in Poland [36], and 16.1% in Iran [37]. However, this method is limited to dairy animals, and there is a growing interest to examine dust samples from animal facilities to detect *C. burnetii* on ruminant farms [3,6,38,39,40,41]. The pathogen was determined in dust samples for more than one year after an outbreak, but information about its infectiousness is limited [3,40]. Viable *C. burnetii* was detected in dust within two months after the last parturition [6]. Windowsills and fences were the preferred sampling locations [3,38,41,42]. Despite the high-risk during milking activity for farmers and farm workers [43,44], milking parlors have been rarely included in studies [45].

The present field investigation aimed to monitor three Q fever outbreaks on dairy goat farms after an inactivated *C. burnetii* phase I vaccine was given. The shedding of *C. burnetii* was analyzed by vaginal swabs and monthly BTM samples over a period of three kidding seasons. Dust samples from one windowsill of each barn and from the milking parlors were included in the investigation to evaluate the intensity and duration of *C. burnetii* contamination of goat facilities. Our clinical long term observations supplement existing statistical models [26] and will contribute to future risk assessments of Q fever outbreaks according to the One Health approach.

## 2. Materials and Methods

### 2.1. Herd History

#### 2.1.1. Dairy Goat Herd A

Dairy goat herd A was located in the German federal state of Schleswig-Holstein (northern Germany) and consisted of 360 does, which had suffered from endemic abortion with 24 aborting goats in January 2018. Later, several stillborn and weak kids were born up until the end of the kidding season in April 2018. Further details about kid losses during this period were not available. In early January 2018, an aborted fetus, including the placenta, was sent to the federal state laboratory of Schleswig-Holstein and examined for abortifacient agents, such as *Brucella* spp., *Campylobacter fetus* ssp. *fetus*, *Chlamydia* spp., *C. burnetii*, *Listeria* spp., and *Salmonella* spp. The only diagnosed pathogenic microorganism was *C. burnetii* (Cq 13; VetMAX™ *C. burnetii* Absolute Quant Kit, Thermo Fisher Scientific GmbH, Dreieich, Germany). All goats were kept in one barn closed on all four sides with wooden walls. Ventilation occurred through the open front and back doors of the barn. The milking parlor was directly connected to the barn but separated by metal fences. The herd was managed semi-intensively under organic farming standards, with access to the pasture during the daytime annually from April until November. The whole dimension of the Q fever outbreak was recently described in detail by the authors [44].

#### 2.1.2. Dairy Goat Herd B

The animal stock of farm B consisted of 152 dairy goats and was located in the German federal state of North-Rhine Westphalia (western Germany). The kidding season took place from February to April 2018 and 20 goats showed reproductive disorders, such as abortion, stillborn, and weak kids at the end of the kidding season. Four aborted fetuses with placentas from two does were examined by the federal state laboratory of North Rhine-Westphalia to detect differential pathogens capable of causing abortion, such as *Brucella* spp., *Campylobacter* spp., *Chlamydia* spp., *C. burnetii*, bluetongue virus, pestivirus and Schmallenberg virus. The only detected abortifacient pathogen was *C. burnetii* (Cq 11–22; VetMAX™ *C. burnetii* Absolute Quant Kit, Thermo Fisher Scientific GmbH, Dreieich, Germany). The barn had three closed sides with wooden walls and one side with barn curtains. The goats had to enter and leave the milking parlor through a flap; therefore, the milking parlor was separated from the barn. On the organic farm, the goats were managed semi-intensively, and grazed during the daytime on pastures close to the barn from April until October each year.

#### 2.1.3. Dairy Goat Herd C

Dairy goat herd C consisted of 85 dairy goats and was located in the German federal state of Bavaria (southern Germany). The main kidding season was in January 2018, but single animals still gave birth until March 2018. In February 2018, the first dead kids were born. In total, six goats gave birth to stillborn or weak kids. Q fever was considered as one cause of the disease, and vaginal swabs were collected from the twelve last kidding goats. The samples were analyzed by qPCR for the presence of *C. burnetii*, and all vaginal swabs tested positive (Cq 25–38; VetMAX™ *C. burnetii* Absolute Quant Kit, Thermo Fisher Scientific GmbH, Dreieich, Germany) by the federal state laboratory of Lower Saxony (Germany). No other disease pathogens were investigated. Three sides of the barn were closed by barn curtains and the fourth side was a brick wall. The milking parlor was separated by a glass door from the barn. The intensively managed goats were mainly kept indoors, but had access to a concreted paddock, which was always accessible from April to November.

All three farmers (A–C) asked the Clinic for Swine and Small Ruminants at the University of Veterinary Medicine Hannover, Foundation, Hannover, Germany for help to combat the Q fever outbreak in their dairy goat herds.

### 2.2. Sample Collection

On farm A, the kidding season was ongoing at the first visit in January 2018, whereas in flock B, only single goats were still pregnant at the first farm visit in April 2018. The main kidding season of herd C had been completed two months earlier when the first measures had been implemented in April 2018.

#### 2.2.1. Blood Samples and Vaginal Swabs

All aborting goats (*n* = 24) from herd A in January 2018 were included, and animals from herd B (*n* = 48) and C (*n* = 35) were randomly selected in April 2018 for serum samples to be taken from the *Vena jugularis* (KABE LABORTECHNIK GmbH, Nümbrecht-Elsenroth, Germany). From these goats, vaginal swabs were also collected at the first farm visit by the authors (B.U.B. and M.G). During the kidding season in 2019 and 2020, the farmers were instructed to carefully collect vaginal swabs from goats within 48 h after parturition. Due to the risk of single ‘super-shedders’ [3,27], as many vaginal swabs as possible were analyzed to describe the *C. burnetii* excretion to the greatest possible extent. Unfortunately, it was not possible to always sample the same goats during the study period because of difficulties in herd management on the farms.

#### 2.2.2. Bulk Tank Milk and Dust Swabs

Bulk tank milk samples were taken monthly to monitor the shedding course at herd level during the entire study period (from first visit until September 2020). In addition, monthly dust samples from the milking parlors and from one windowsill of each barn were collected, as described previously [3]. Briefly, a dry swab (Sarstedt AG & Co. KG, Nümbrecht, Germany) was rolled over the same windowsill from each barn covering one meter to collect dust. The same procedure was performed to collect dust from the milking parlor. The swab was rolled over the milk and vacuum pipeline (farm A) of the milking parlor on farm A or over one windowsill, which was located in the room of the milking parlors on farms B and C. The windowsills were chosen for sampling because the milk and vacuum pipelines were not easily accessible on farms B and C. The sample locations within the barns and milking parlors were always the same on each farm. Neither cleaning nor disinfection was performed in any of the three barns and milking parlors during the entire study period.

### 2.3. Vaccination Schedules and Breeding Management

All goat herds were vaccinated for the first time with an inactivated *C. burnetii* phase I vaccine (Coxevac^®^, Ceva, Libourne, France) in accordance with the manufacturer’s instructions. Goats in herds B and C were annually revaccinated and the female progeny received their primary vaccination four weeks before mating. Animals in herd A had already been boosted in September 2018 before the breeding season started. This also included the primary vaccination of the female offspring from 2018. Since 2019, only the young offspring were vaccinated twice before breeding but the multiparous goats were left untreated on farm A. This modified vaccination schedule was performed due to financial reasons. In addition, all farmers were urged to remove aborted fetuses and afterbirth from the straw beddings as soon as possible and to store these materials in containers until disposal through rendering plants. The animal facilities on all three farms were neither cleaned nor disinfected during the entire study period.

Farms A and B continuously milked approximately half of the herd after the Q fever outbreak in 2018. The remaining does were mated and dried-off around six weeks before kidding. Farm C bred the entire herd with a dry-off period of six weeks. Therefore, BTM samples were not always available from herd C.

An overview of the timing of blood/vaginal swab sampling, kidding seasons, and vaccination in all three dairy goat herds is provided in Supplementary Figure S1.

### 2.4. Laboratory Analysis

Goat sera were examined with two phase-specific ELISAs (EUROIMMUN AG, Lübeck, Germany) in accordance with the manufacturer’s instructions and this process has recently been described [46]. The test results were presented quantitatively in relative units (RU) determined by a standard curve. The manufacturer specified serum samples with RU ≥ 22 as positive.

*Coxiella burnetii*-specific DNA fragments in the vaginal swabs were detected by amplification of the IS*1111* elements with qPCR. Cycle Quantification (Cq) values ≤ 45 were indicated as positive and Cq values >45 as negative values according to Frangoulidis and colleagues [47]. The BTM and dust samples were examined with a commercially available qPCR (LSI VetMAX^TM^ *C. burnetii* Absolute Quant Kit, Thermo Fisher Scientific GmbH, Dreieich, Germany) targeting IS*1111* as well. The manufacturer indicated Cq values ≤ 45 as positive.

### 2.5. Statistical Analyses

Serum values were checked for normal distribution by the Shapiro–Wilk-Test, followed by a *t*-Test or Mann–Whitney-Test to compare the phase-specific IgG response within a goat herd. Results with *p* < 0.05 were considered to be significant.

The vaginal swabs were evaluated using descriptive methods. Both the amount of *C. burnetii* DNA and the number of positive vaginal swabs were considered.

The statistical software SAS (SAS Institute Inc., Cary, NC, USA) was used for all calculations.

## 3. Results

### 3.1. Serology

**Figure 1:**
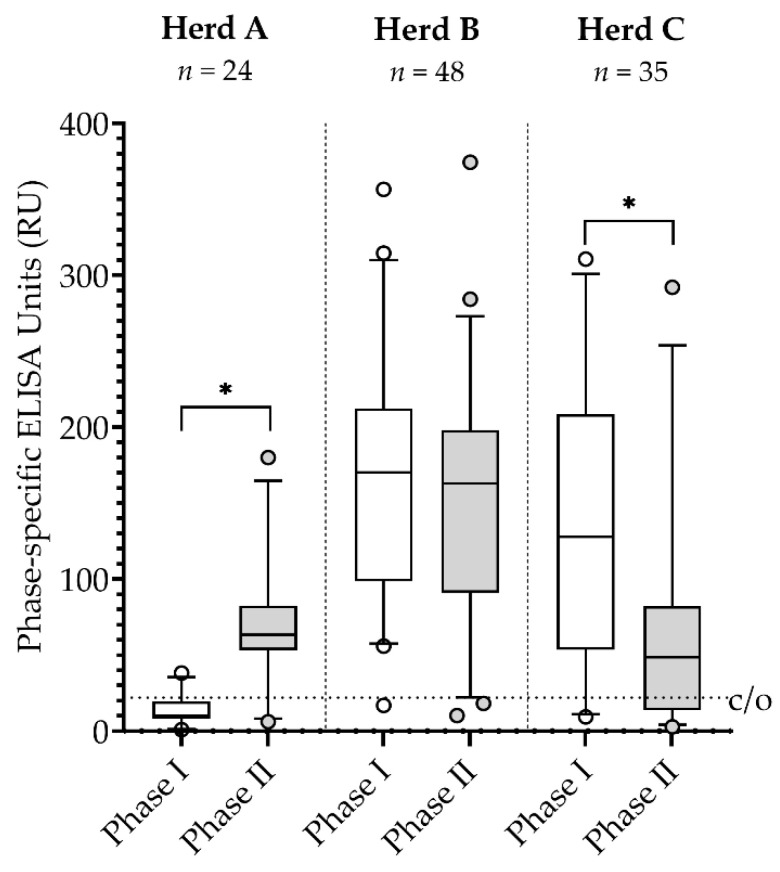
Median levels of IgG phase I and phase II *Coxiella burnetii* antibodies in three naturally infected dairy goat herds (A–C) at the start of the investigation in 2018. * *p* < 0.05; c/o = ELISA cut-off.

In goat herd A, the median level of IgG PhII antibodies was significantly higher than the IgG PhI antibodies (Figure 1). The phase-specific antibodies did not significantly differ in herd B, and the IgG PhI median level was significantly higher than the IgG PhII level in herd C.

### 3.2. Vaginal Swabs

All 24 examined goats from herd A shed *C. burnetii* in 2018, and half of the vaginal swabs contained large amounts of *C. burnetii* DNA (Cq ≤ 20). At the subsequent kidding seasons, goats still shed small quantities (Cq ≥ 33) of *C. burnetii*.

In herd B, all vaginal swabs tested *C. burnetii* positive at the initial sampling date (Cq ≤ 33). In 2019 and 2020, the number of vaginal shedders decreased, and vaginal swabs contained small amounts of *C. burnetii* DNA (Cq ≥ 37).

In 2018, 25 of 35 goats shed small amounts (Cq ≥ 34) of *C. burnetii* through the vaginal route in herd C. During the following kidding seasons, fewer does excreted small quantities of *C. burnetii* (Cq ≥ 36).

**Figure 2:**
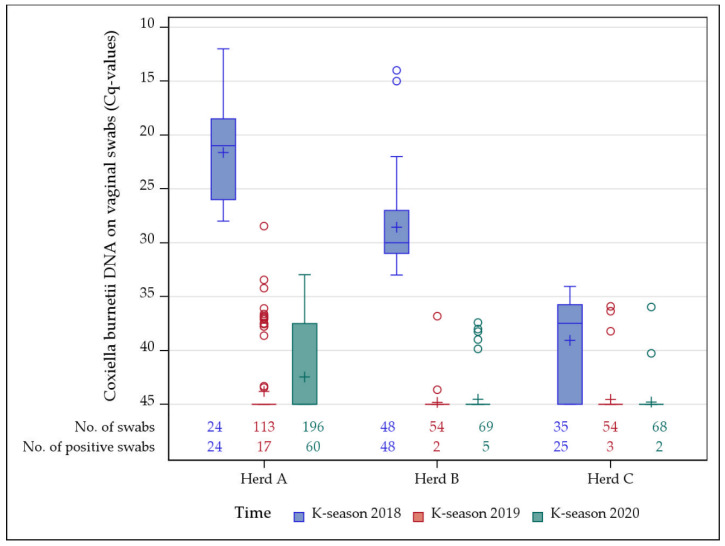
*Coxiella burnetii* shedding through vaginal route during three (2018–2020) kidding seasons (K-season) in three naturally *C. burnetii* infected dairy goat herds (A–C) analyzed by qPCR. All goat herds were vaccinated after *C. burnetii* had been diagnosed in 2018.

Details of the number of vaginal shedders and *C. burnetii* DNA amount (Cq-values) on vaginal swabs are shown in Figure 2.

### 3.3. Bulk Tank Milk

**Figure 3:**
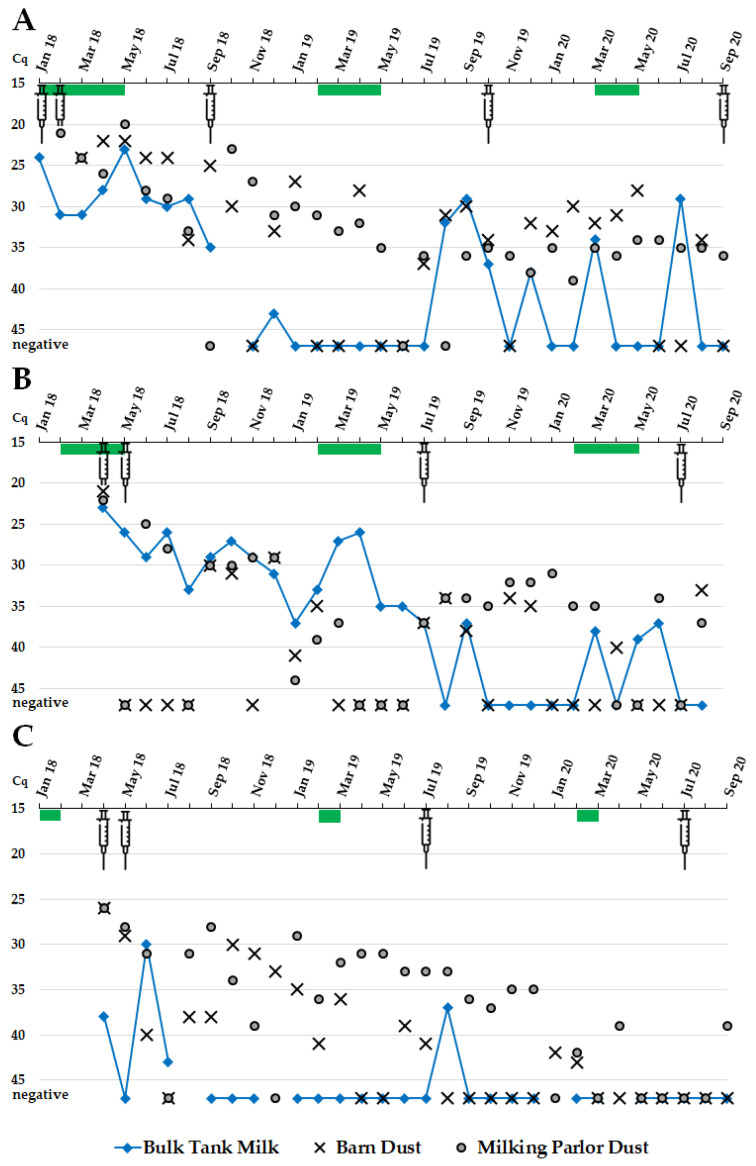
*Coxiella burnetii* detection by qPCR (Cq ≤ 45: positive result) in monthly collected bulk tank milk specimens ◆ and dust samples from one windowsill of each barn ✖ and from each milking parlor ● in three naturally *C. burnetii* infected and vaccinated dairy goat herds (**A**–**C**). BTM samples were not always available at every sampling date from farm A and C. Green bar: kidding period; syringe: vaccination; missing samples are not indicated.

After the initial detection of *C. burnetii* in the dairy goat herds, pathogen DNA was continuously detected in BTM samples for 9 and 16 months in herds A and B, respectively. In the following months, *C. burnetii* was identified irregularly in the BTM. In contrast, BTM specimens from goat herd C tested *C. burnetii* positive at the beginning of the investigations and once again in August 2019 (Figure 3). In total, 50% (16/32), 69% (20/29), and 15% (4/26) positive BTM samples were detected in herds A, B, and C, respectively.

### 3.4. Dust from Barns and Milking Parlors

Dust samples from the barns and milking parlors tested discontinuously *C. burnetii* positive on all three goat farms (Figure 3). On farm A, 71% (22/31) of the barn dust samples tested *C. burnetii* positive, whereas specimens from farms B and C had 45% (13/29) and 50% (15/30) positive outcomes. The number of positive dust samples was higher from the milking parlors; 91% (A, 29/32), 72% (B, 21/29), and 73% (C, 22/30), respectively, compared to the windowsills from each of the barn. The *C. burnetii* burden from both sample locations showed an undulating development.

## 4. Discussion

In the past, *C. burnetii* shedding small ruminants have caused small-scale human Q fever outbreaks [2,12] in Germany, and one family member from farm A developed acute Q fever [44]. Without countermeasures, *C. burnetii* circulates in goat flocks for a certain time [24,26,48]. One animal can shed large amounts of the pathogen, leading to a high number of human Q fever cases [12]. So-called ‘super-shedders’ were also detected in herds A and B in 2018. Therefore, we did not include control animals for vaccine evaluation due to public health reasons. Moreover, non-vaccinated animals within a positive herd increase the pathogen exposure and this may lead to underestimation vaccine efficiency [30,49]. In addition, such an approach does not support the overall objective to control Q fever outbreaks. Information about fully vaccinated goat herds after a Q fever outbreak is lacking, but is essential to help decision-makers of public health authorities to implement or lift restrictions. Therefore, our observations are in line with the One Health approach of improving the understanding of fully vaccinated goat herds after Q fever outbreaks.

The examination of goat sera with phase-specific ELISAs revealed different Q fever disease stages among all three dairy goat herds. According to the serological results with a dominance of IgG Ph II antibodies, the dairy goat herd A suffered from an acute Q fever outbreak. This is underlined by the high amount of *C. burnetii* excretion by half of the examined animals (Cq ≤ 20) in January 2018. In herd B, an ongoing infection was revealed with similar levels of both phase-specific IgGs, which coincides with the sampling time right after the kidding season, as the acute disease was already over. In contrast, the median of IgG Ph I antibodies was significantly higher compared to IgG Ph II in herd C, thus suggesting an infection in the past. This interpretation is supported by the fact that the samples were collected around two months after the main kidding season had been completed, and only 71% of vaginal swabs tested positive with a low *C. burnetii* amount (Cq ≥ 34). Our findings are consistent with outcomes from naturally or experimentally infected goats [22,23,24]. Consequently, with the new phase-specific ELISAs, classification of *C. burnetii* positive goat herds into three different stages of infection was possible. Hence, the assays are helpful tools for evaluating the disease status in practice.

The reasons for the lack of control groups were already discussed in detail above. Therefore, we can only speculate about the effect of the vaccine on vaginal shedding. Nevertheless, taking into account that a considerable number of non-vaccinated goat herds still shed *C. burnetii* during the following kidding seasons [48,50], we suspect a positive effect of the vaccine to reduce the number of vaginal shedders and amount of pathogen excretion at subsequent parturitions. In all herds, the vaccine could not prevent shedding during the subsequent kidding periods, which is in line with previous findings [3,29,30,31]. Taken together, our clinical results indicate that vaccination for three years is insufficient to eliminate *C. burnetii* from infected dairy goat herds and emphasize the necessity for annual Q fever vaccination for at least six years, as claimed by simulation models [26].

The irregular detection of *C. burnetii* DNA in BTM samples from herds A and B suggests intermittent milk shedders [51], which have so far only been confirmed in cattle [52,53] and not in goats. The risk of acquiring Q fever by drinking raw milk or consuming raw milk products is low but cannot be fully excluded. In addition to *C. burnetii*, infected goats can also shed other zoonotic pathogens through milk, such as *Toxoplasma gondii*, *Brucella melitensis*, *Listeria monocytogenes,* and tick-borne encephalitis virus [54,55,56,57]. Due to the increasing popularity of drinking raw milk and new routes of sale for raw milk through vending machines and internet sale [58], it is necessary to raise the awareness of food-borne pathogens in raw milk and raw milk products.

Removing goats shedding *C. burnetii* intermittently in milk, led to negative BTM samples [51]. However, the ‘test and cull’ approach is cost-intensive and, thus, difficult to enforce in practice. Moreover, it will not result in eradicating Q fever if applied as the exclusive control strategy [26]. In addition, vaccinating dairy goats with an inactivated *C. burnetii* phase I vaccine caused positive milk samples by qPCR up until nine days after vaccination [59]. Shedding of vaccine-antigen-DNA might be the reason for a single positive BTM sample (Cq 37) during 2019 in herd C, 31 days after the booster vaccination. Therefore, timing the vaccination and testing have to be considered if BTM samples are used for Q fever surveillance.

The dust from the barns and milking parlors of all three dairy goat farms contained considerable amounts (Cq < 30) of *C. burnetii* after the initial detection of the pathogen in 2018. The severe contamination is associated with the tremendous release of *C. burnetii* from aborting or kidding goats [3,6,38]. During the following months, the detection of *C. burnetii* from dust samples had an undulating development. Several factors affect the probability of detecting *C. burnetii* in animal facilities, such as sampling method and matrix, sampling location, used PCR method, activities during sampling, history of abortion, number of reproductive females, number of vaginal shedders, ventilation, and type of ruminant species [3,38,40,41,42,45,60,61]. This highlights the need for a standardized sampling method and performance to make the outcomes reliable and comparable.

Dust samples from the milking parlors were most frequently *C. burnetii* positive on all three dairy goat farms. Inhalable dust particles are raised during milking activity [45]. Hence, people performing daily milking are at a high risk of becoming infected with *C. burnetii* [43,44]. This should be taken into account in future risk assessments for farmers and farm workers. However, the detection of *C. burnetii* DNA from the surroundings provides no information about the viability of the pathogen. It is assumed that *C. burnetii* remains infectious in dust for up to two months after parturition [6]. Finally, the infectiousness of low *C. burnetii* DNA amounts in the environment remains doubtful and needs further clarification. Additionally, the barns and the milking parlors were contaminated for more than two years on all three farms. This is in line with observations from other studies on small ruminant farms [3,40]. Therefore, our findings point out the need for efficient sanitation protocols to decontaminate animal facilities in order to prevent pathogen spillovers to humans.

The authors are aware of the limitations of the present field study. Differences in stock density, interior design of barns and milking parlors, ventilation, management, and vaccination strategies, as well as *C. burnetii* infection status make a direct comparison among the three dairy goat herds difficult and limit the interpretation of our longitudinal study. In addition, it must be taken into account that *C. burnetii* strains on the three farms might contain different numbers of IS*1111* elements [62]. Therefore, the results of the qPCR between the three farms are also not comparable. All these variations highlight the challenges in monitoring and controlling Q fever outbreaks in veterinary practices.

## 5. Conclusions

Phase-specific serology enables the classification of the Q fever disease status of goat herds and supports the identification of acute cases, which are a serious hazard for humans. The inactivated *C. burnetii* phase I vaccine did not prevent vaginal shedding in naturally pre-infected dairy goats at two subsequent kidding seasons, and underlines the need for vaccinating *C. burnetii* positive dairy goat herds for at least six years [26]. The long-term detection of *C. burnetii* in dust samples from milking parlors emphasizes the threat during milking activities. In the future, routine methods to determine the infectiousness of low *C. burnetii* amounts in dust samples are urgently needed to assess the risk of infection for humans and animals. In addition, the sensitivity of dust swabs to identify *C.* *burnetii* positive livestock has to be evaluated. Altogether, our observations highlight the use of phase-specific serology and the molecular analysis of various sampling matrices to monitor the status and shedding of *C. burnetii* in goat herds. The generated data are extremely helpful for Q fever risk assessments and supports the One Health approach.

## Data Availability

The data are available on request from the corresponding author.

## Supplementary Materials

The following supporting information can be downloaded at: https://www.mdpi.com/article/10.3390/vetsci9030102/s1, Figure S1: Overview of blood/vaginal swab sampling, kidding seasons and vaccination schedules in three *Coxiella burnetii* positive dairy goat herds (A–C).
