# Supplementary material for: Surveillance of Coxiella burnetii Shedding in Three Naturally Infected Dairy Goat Herds after Vaccination, Focusing on Bulk Tank Milk and Dust Swabs
Source: Vet Sci. 2022 Feb 24;9(3):102. doi: 10.3390/vetsci9030102 (PMC8950187; doi:10.3390/vetsci9030102)
Supplement: Supplementary file 1 [file vetsci-09-00102-s001.zip › SupplementaryFigure1.pdf]

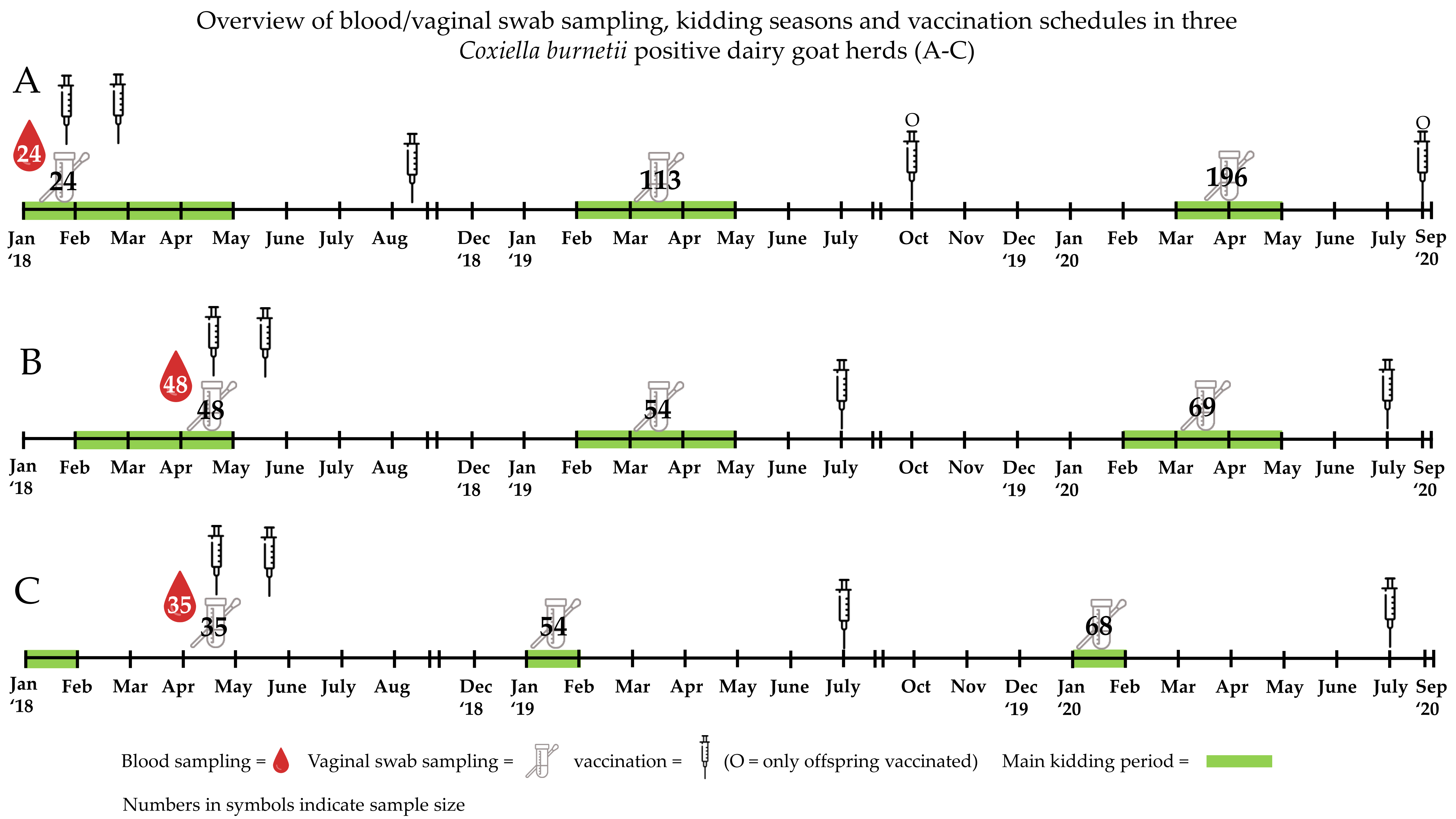
Overview of blood/vaginal swab sampling, kidding seasons and vaccination schedules in three
Coxiella burnetii positive dairy goat herds (A-C)
A
24
24
113
196
O
O
Jan '18
Feb
Mar
Apr
May
June
July
Aug
Dec '18
Jan '19
Feb
Mar
Apr
May
June
July
Oct
Nov
Dec '19
Jan '20
Feb
Mar
Apr
May
June
July
Sep '20
B
48
48
54
69
Jan '18
Feb
Mar
Apr
May
June
July
Aug
Dec '18
Jan '19
Feb
Mar
Apr
May
June
July
Oct
Nov
Dec '19
Jan '20
Feb
Mar
Apr
May
June
July
Sep '20
C
35
35
54
68
Jan '18
Feb
Mar
Apr
May
June
July
Aug
Dec '18
Jan '19
Feb
Mar
Apr
May
June
July
Oct
Nov
Dec '19
Jan '20
Feb
Mar
Apr
May
June
July
Sep '20
Blood sampling =
Vaginal swab sampling =
vaccination =
(O = only offspring vaccinated)
Main kidding period =
Numbers in symbols indicate sample size
